# Neuromorphic artificial intelligence systems

**DOI:** 10.3389/fnins.2022.959626

**Published:** 2022-09-14

**Authors:** Dmitry Ivanov, Aleksandr Chezhegov, Mikhail Kiselev, Andrey Grunin, Denis Larionov

**Affiliations:** ^1^Cifrum, Moscow, Russia; ^2^Faculty of Computational Mathematics and Cybernetics, Lomonosov Moscow State University, Moscow, Russia; ^3^Faculty of Physics, Lomonosov Moscow State University, Moscow, Russia; ^4^Laboratory of Neuromorphic Computations, Department of Physics, Chuvash State University, Cheboksary, Russia

**Keywords:** neuromorphic computing, brain-inspired computing, neuromorphic, neuromorphic accelerator, memristor, neural network, AI hardware

## Abstract

Modern artificial intelligence (AI) systems, based on von Neumann architecture and classical neural networks, have a number of fundamental limitations in comparison with the mammalian brain. In this article we discuss these limitations and ways to mitigate them. Next, we present an overview of currently available neuromorphic AI projects in which these limitations are overcome by bringing some brain features into the functioning and organization of computing systems (TrueNorth, Loihi, Tianjic, SpiNNaker, BrainScaleS, NeuronFlow, DYNAP, Akida, Mythic). Also, we present the principle of classifying neuromorphic AI systems by the brain features they use: connectionism, parallelism, asynchrony, impulse nature of information transfer, on-device-learning, local learning, sparsity, analog, and in-memory computing. In addition to reviewing new architectural approaches used by neuromorphic devices based on existing silicon microelectronics technologies, we also discuss the prospects for using a new memristor element base. Examples of recent advances in the use of memristors in neuromorphic applications are also given.

## 1. Introduction

Modern AI systems based on neural networks would not be possible without hardware that can quickly perform a large number of repetitive parallel operations. Modern AI systems have become possible and gained widespread use due to hardware and large datasets. As shown in Hooker (2020), throughout the AI history, precisely those approaches won for which there was suitable hardware. That is why it is important to consider *AI algorithms in conjunction with the hardware that they run on*. It is the hardware that determines the availability and effectiveness of AI algorithms.

In this article, we briefly describe the main principles of modern AI systems based on the von Neumann architecture and classical neural networks and highlight their drawbacks. Then we discuss neuromorphic systems and various approaches to their implementation by mimicking some features of the brain.

It is worth noting that the term “neuromorphic”, introduced by Caver Mead in his pioneering works (Mead, 1990; Douglas et al., 1995), originally meant analog VLSI circuits focused on emulating the behavior of neural systems. But today this term encompasses various computing systems in which the principles of organization and working mechanisms are inspired by the biological brain (Zhang et al., 2020; Frenkel et al., 2021a).

### 1.1. Related works

There are several reviews of neuromorphic approaches (e.g., Schuman et al., 2017; Zhang et al., 2020; Frenkel et al., 2021a; Shrestha et al., 2022).

In Shrestha et al. (2022), the authors consider digital/mixed-signal implementations of Spiking Neural Networks (SNNs) and propose to classify neuromorphic systems on the model of neurons they support, implementation choice (analog, digital, mixed), architecture choice and network on chip principles of organization.

In Zhang et al. (2020), the authors consider neuromorphic systems that support both Artificial Neural Networks (ANNs) and SNNs. They propose four key metrics to compare systems: compute density, energy efficiency, compute accuracy, and on-chip learning capability.

The authors of Schuman et al. (2017) present a review of over 3,000 papers covering the 35-year history of neuromorphic computations. They consider the main motivations for neuromorphic computing (e.g., parallelism, von Neumann bottleneck, scalability, low power consumption, etc.). Also they review neuro-inspired models, learning algorithms, hardware, and applications.

In Frenkel et al. (2021a), the authors review the neuromorphic field and classify all neuromorphic systems into two approaches: top-down and bottom-up. Systems in the top-down approach try to reproduce natural intelligence. Systems from the bottom-up approach attempt to solve practical AI problems. For both groups the authors consider their silicon implementations, necessary algorithms and models.

An important distinction of the present review is the consideration of neuromorphic systems from viewpoint of their *proximity to the properties of biological neuronal systems*. This may be an insightful aspect for analysis of modern and the development of future neuromorphic systems.

### 1.2. von Neumann architecture

A great majority of the latest AI systems are built by pairing von Neumann computers and classical neural networks, dating back to the Rosenblatt's perceptron.

The von Neumann architecture separates the memory and the computations (Hennessy and Patterson, 2017). The computations are executed in the form of programs, which are sequences of machine instructions. Instructions are performed by a processor. A processor instruction usually has several arguments that it takes from processor registers (small but very fast memory cells located in the processor). At that, the instructions and most of the data are stored in the memory separately from the processor. The processor and the memory are connected by a data bus by which the processor receives instructions and data from the memory.

The first bottleneck of this architecture is the limited throughput of the data bus between the memory and the processor. During the execution of a program, the data bus is loaded mainly by the transfer of processing data from/to Random Access Memory (RAM). Moreover, the maximum throughput of the data bus is much less than the speed at which the processor can process data.

**Figure 1:**
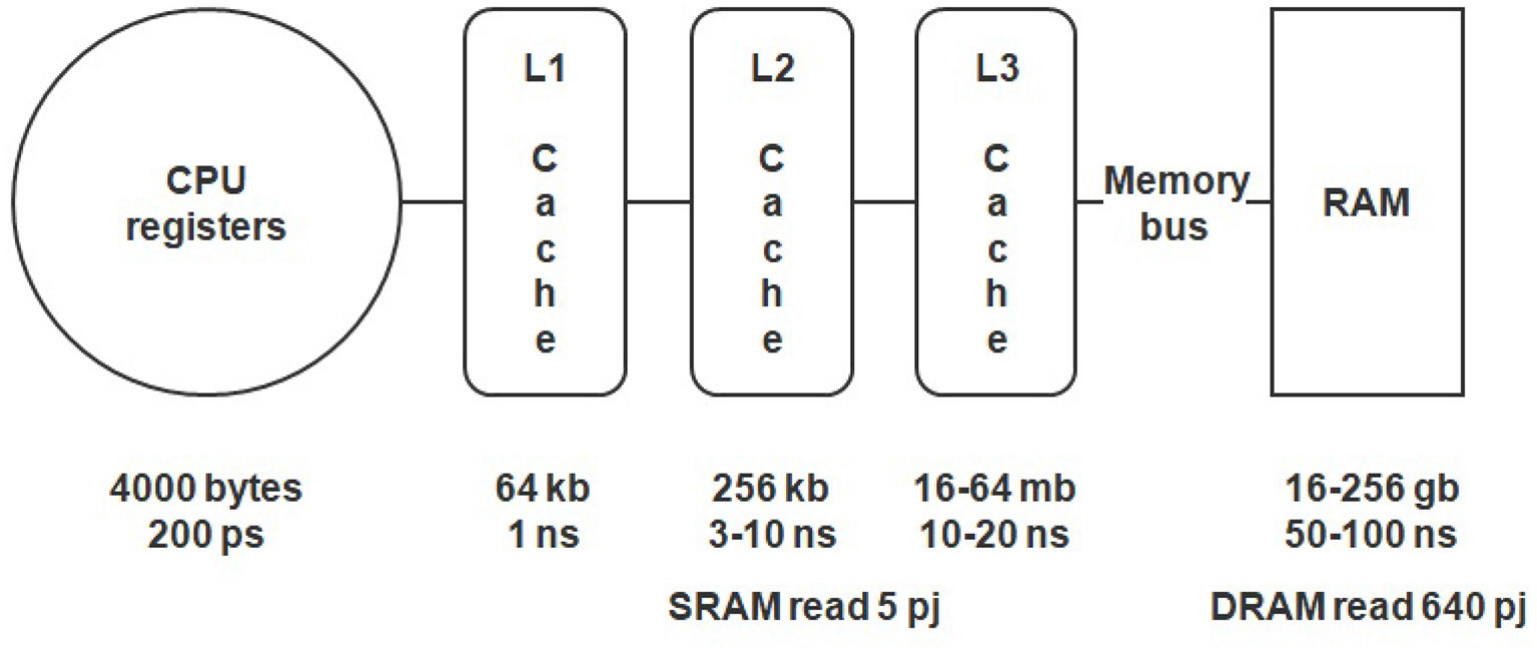
Memory hierarchy, access speed, and power consumption of a CPU.

Another important limitation is the big difference in the speed of RAM and processor registers (see Figure 1). This can cause latency and processor downtime while it fetches data from the memory. This phenomenon is known as the von Neumann bottleneck.

It is also worth noting that this approach is energy-intensive. As argued in Horowitz (2014), the energy needed for one operation of moving data along the bus can be 1,000 times more than the energy for one computing operation. For example, adding two 8 bit integer numbers consumes ~0.03 pJ while reading from Dynamic Random Access Memory (DRAM) consumes ~2.6 nJ.

### 1.3. Neural networks based on the von Neumann architecture

To solve cognitive problems on computers, there was developed the concept of ANN based on the perceptron and the backpropagation (Rumelhart et al., 1986) method (Goodfellow et al., 2016). Perceptron is a simplified mathematical model of an artificial neuron network, in which neurons compute a weighted sum of their input signals and generate an output signal using an activation function. The process of training the network by the backpropagation method consists of modifying its weights toward decreasing the error (loss function).

**Figure 2:**
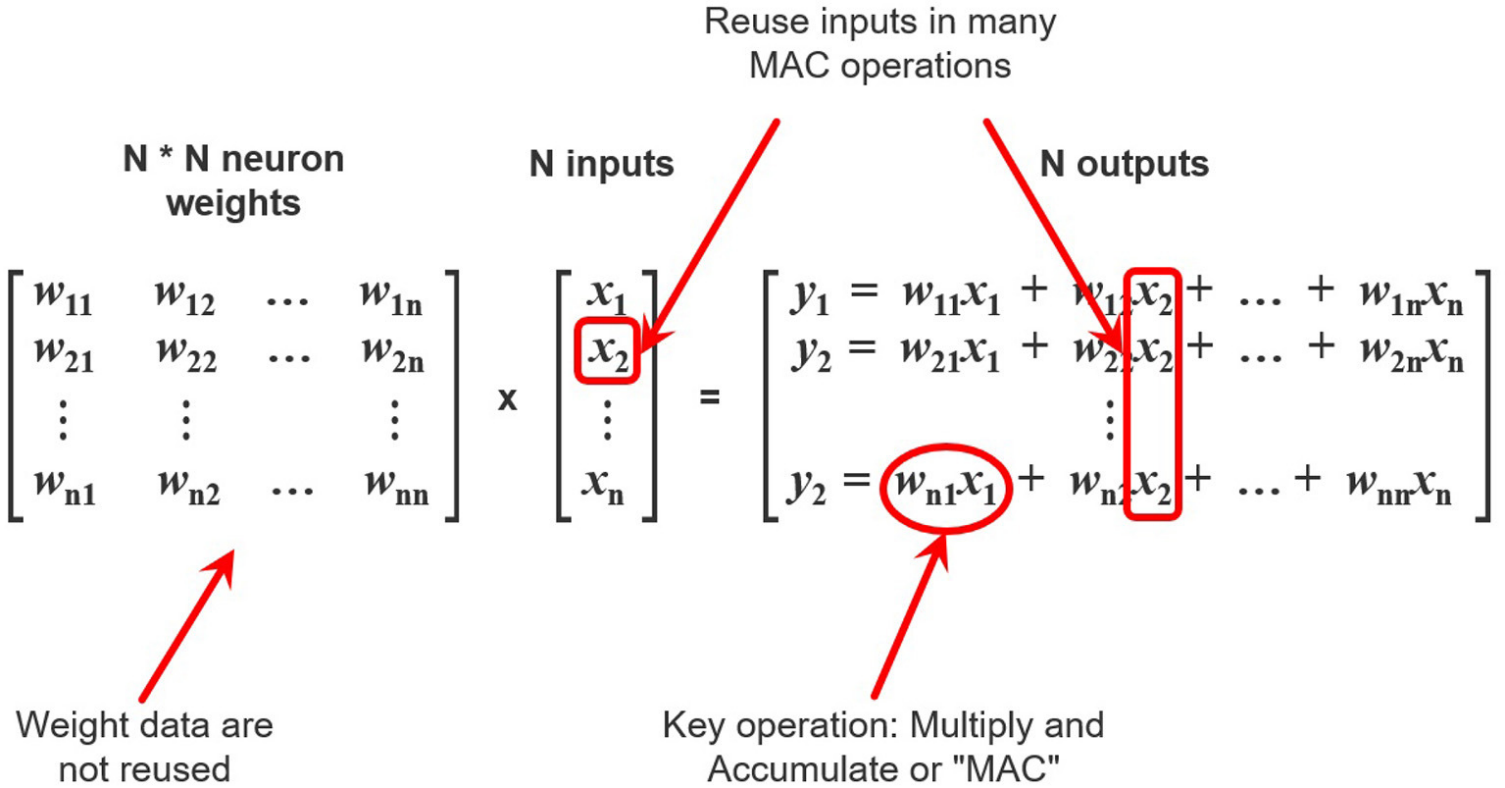
A schematic view of neural network computations. Elements of input vector x are reused n times while weights *w*_*ij*_ are used once.

Since the majority of modern neural networks have a layered architecture, the most computationally intensive operation in these networks is the operation of multiplying a matrix by a vector *y* = *Wx*. To carry out this operation, it is first necessary to obtain data from the memory, namely, m*n weights of *W* and *n* values of vector *x*. It should be noted that m*n weights will be used once per matrix-vector multiplication while the values from the vector *x* will be reused (Figure 2).

Thus, in order to perform computations, the processor needs to receive weights and input data from the memory. As mentioned above, the throughput of the data bus and the latency in receiving data limit the speed of obtaining weights. Also, the number of weights grows as *O*(*n*^2^), where n is the input size. However, there is the limit of the throughput of the data bus, connecting the processor with the memory and transferring the weights and the input vectors. It becomes exhausted much earlier than the available amount of computation (multiplying matrices by a vector and applying activation functions) per unit time.

### 1.4. Mitigating limitations in modern computing systems

First, let us look at the ways to mitigate the above limitations in modern AI systems.

#### 1.4.1. Central processing unit

Classically, the problem of memory latency was solved in the Central Processing Unit (CPU) by using a complex multi-level cache (a high-speed data storage that speeds up access to data) system (see Figure 1) (Hennessy and Patterson, 2017). In modern processors, caches can occupy up to 40% of the chip area, providing tens of megabytes of ultra-fast memory. Usually, the size of practically used neural networks does not allow to fit all the weights into caches. Nevertheless, the latest processors with vertically stacked caches technology (e.g., AMD Ryzen 7 5800X3D) can change this situation, providing larger caches.

Other traditional approaches to CPU optimization were speculative execution, branch prediction, and others (Hennessy and Patterson, 2017). However, in matrix multiplication, the order of computations is known in advance and does not require such complex approaches, which makes these mechanisms useless. This means that in the field of ANNs the CPU can only be suitable for computing small neural networks, and it is unsuitable for modern large architectures hundreds of megabytes in size.

#### 1.4.2. Graphical processing unit

The Graphical Processing Unit (GPU) is a massively parallel architecture. It consists of a large number of computing cores combined into streaming multiprocessors. This allows to execute single instruction thread over multiple data streams (SIMD thread). Moreover GPU executes several SIMD threads.

**Figure 3:**
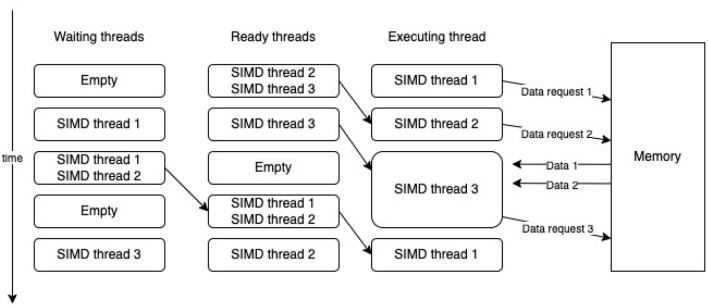
Switching between SIMD threads.

The GPU uses several strategies to deal with memory latency. The main one is to give each streaming multiprocessor a large register file that saves the execution context for many threads and provides quick switching between them. The computation scheduler uses this feature and, when an instruction with a high latency is executed in one of the instruction threads [Single Instruction Multiple Data (SIMD) thread 1], for example, obtaining data from memory, it immediately switches to another instruction thread (SIMD thread 2), and if a latency occurs in this additional thread, the scheduler begins a new ready instruction thread (SIMD thread 3). After some time, data for the first thread arrives and it also becomes ready for execution (see Figure 3). This enables memory latency to be hidden (Hennessy and Patterson, 2017).

However, what is crucial aside from latency is the memory throughput, i.e., the maximum amount of data that can be received from the memory per unit time. To solve this problem in GPUs, NVIDIA began adding High Bandwidth Memory (HBM) starting with the P100 (2016), and this dramatically increased their performance compared to previous generations. In the Volta and Turing architectures, Nvidia continued to increase the memory throughput, bringing it up to 1.5 TB/s in the A100 architecture (Krashinsky et al., 2020).

#### 1.4.3. Tensor processing unit

Google announced the first Tensor Processing Unit (TPU)—TPUv1 in 2016 (Jouppi et al., 2018). It mitigates latency and low memory throughput by using so-called systolic matrices and software-controlled memory instead of caches. The idea of systolic computations is to create a large matrix (256 × 256 for TPUv1) of computing units. Each unit stores a weight and performs two operations. First, it multiplies number x that has come from the unit above by the weight and adds the result to the number that came from the unit to the left. Second, it sends the number x received from above to the unit below, and forwards the received sum to the unit to the right. This is how the TPU performs matrix multiplications in a pipeline. With a sufficiently large batch size, it will not have to constantly access the weights in memory, since the weights are stored in the computing units themselves. At that, having a batch size larger than the width of the systolic array, the TPU will be able to produce one result of multiplying a 256x256 matrix by a 256-long vector each cycle.

#### 1.4.4. Neuromorphic approach

Despite significant advances and market dominance of the hardware discussed above, the AI systems based on them are still far from their biological counterparts. There is a gap in the level of energy consumption, flexibility (the ability to solve many different tasks), adaptability and scalability. However, such problems are not observed in the mammalian brain. In this connection, it can be assumed that, as it already happened with the principle of massive parallelism (Rumelhart and McClelland, 1988), the implementation of crucial properties and principles of the brain operation could reduce this gap. As a response to this need, a neuromorphic approach to the development of AI systems attempts to use of the principles of organization and functioning of the brain in computing systems.

The brain is an example of a fundamentally different, non von Neumann, computer. Unlike classical neural networks executed in modern computing systems, in the brain:

- The power consumtion of the human brain is only tens of watts. This is in orders of magnitude less then the consumption of modern AI systems.
- Neurons exchange information using discrete impulses, i.e., spikes.
- All events are transmitted and received asynchronously—there is no single process that explicitly synchronizes the work of all neurons.
- Learning processes are local and network topologies are non-layered.
- There is no common memory that universal processors work with. Instead, a large number of simple computational cells (neurons) function in a self-organizing manner.

## 2. Neuromorphic approaches in computing systems

Today, many kinds of neural network accelerators are arguably misclassified as neuromorphic AI systems to attract attention. To reduce the degree of uncertainty in classifying AI systems as neuromorphic, we propose a list of neuromorphic properties that appear to be useful in creating computing systems and have proven themselves in real-life projects:

- Connectionism: the capability of learning (no need to set the parameters explicitly), the emergence of intellectual properties by linking a large number of relatively simple elements into a network.
- Parallelism: parallel work of neurons, simultaneous execution of different tasks.
- Asynchrony: no single synchronizing process.
- Impulse nature of information transmission: minimum overhead for signal transmission and signal processing at the receiving neuron, resistance to noise.
- On-device learning: the ability of continuous and incremental learning.
- Local learning: lower overhead for data transfer operations during learning, the ability to create unlimitedly large systems.
- Sparsity: event-driven signal processing, the lower overhead for data transfer and data processing.
- Analog computing: efficient hardware implementation.
- In-memory computing: no overhead for transferring intermediate data, no competitive memory access.

Let us consider neuromorphic properties in more detail in the following sections.

### 2.1. Connectionism

The central idea of connectionism is that mental phenomena can be described in terms of Neural Networks (NN) (Buckner and Garson, 2019).

NNs are computing systems inspired by biological neural networks. They consist of a large number of simple units, which are models of biological neurons with varying degrees of similarity. These units are interconnected together with weights, which are artificial analogs of the synapses linking neurons between each other. Any mental state in NN is a vector of activations over neurons.

The neural network learns by finding the appropriate weights, which makes it possible to solve certain problems. It was shown in a big number of experiments that NNs have the ability to learn different skills, such as pattern recognition, language modeling, playing computer games (Goodfellow et al., 2016), etc.

The ability to solve a specific problem and the quality of its solution can be determined by both the neuron model and the network topology.

### 2.2. Parallelism

Each biological neuron is an independent computer, but much slower than modern silicon processors. However, the number of biological neurons in the brain that perform coordinated work reaches 87 billion. Back in the late 1980s, researchers (Rumelhart and McClelland, 1988) came to the conclusion that massively parallel architectures would be required for the efficient operation of neural networks. In support of this idea, it was the massive use of the highly parallel architectures (mostly GPU) that had ensured the current success of neural networks.

### 2.3. Asynchrony

However, when synchronization between computing nodes is required, parallelism by itself does not always give the desired computing effect. According to Amdahl's law (Rodgers, 1985; Bryant and O'Hallaron, 2015), the synchronization overheads grow non-linearly as the number of computing nodes increases, thus limiting the gain from parallelism. Moreover, synchronization consumes power. For example, in modern synchronous digital circuits the clock tree, that distributes the clock signal inside a circuit consumes 20–45% of the total power consumption of the chip (Frenkel et al., 2021a). But the brain does not seem to have a mechanism that explicitly synchronizes the work of all neurons. Biological neurons work asynchronously, which makes it possible to bypass the limitations of Amdahl's law and avoid power consumption overhead for the propagation of a synchronization signal.

### 2.4. Impulse nature of information transmission

In the brain information is transmitted in the form of nerve impulses (Sterling and Laughlin, 2015; Miller, 2018), i.e., abrupt, short changes in potential that travel along nerve fibers and always have approximately the same duration and amplitude. SNN (Maass, 1997) is a popular mathematical model that describes the impulse nature of the information. In SNNs, neurons exchange spikes, i.e., elementary events that have no attributes other than the time of their generation. In that, the transmission of a spike from neuron to neuron does not occur instantly, but requires some time that varies for different pairs of neurons. Thus, each synapse can be characterized not only by the weight but also by the time delay. Spike times and delays serve as a mechanism for explicitly introducing time into the computing model.

Information transmission in the form of impulses appears to have key advantages as compared to the transmission of real numbers, used in traditional neural networks:

- Data can be transferred between neurons in a simple asynchronous way,
- SNNs make it possible to work with dynamic data, as it explicitly includes a time component. In SNN, information is encoded based on the time of spike generation and the presence of a delay in spike propagation from neuron to neuron,
- SNN is a complex non-linear dynamic system,
- It is energy efficient. The activity of a neuron is reduced to its reaction to the arriving spike, hence after this reaction is complete, the neuron goes into an inactive state that consumes only small amount of energy (Niven, 2016). Thus, at each moment of time only a small part of neurons in the network is in the “active mode” and consumes energy.

However, today we see only a few SNN applications in practical tasks. In addition to difficulties with hardware, classical algorithms still outperform SNNs in terms of the quality of problem solving. Despite a large number of academic papers demonstrating solutions of simple problems, SNN training and SNN topologies remain open challenges.

### 2.5. On-device learning

Many AI systems, especially Edge AI systems, are able only to work in inference mode (Zhang et al., 2020). On the other hand the human brain is able to continuously learn. Thus the on-device learning seems to be an important feature of neuromorphic systems. On-device (on-chip) learning is necessary to customize and personalize smart devices according to the needs of the user and enhances privacy by avoiding the transmission of user data to the cloud (Frenkel et al., 2021a).

### 2.6. Local learning

The training of classical neural networks is based on the backpropagation algorithm, which is a special case of the gradient descent method (Rumelhart et al., 1986). The use of gradient descent methods in the brain is hardly realistic because it would be necessary to apply a corrective signal to each neuron, that is computed somewhere based on the results of the network. This means that a feedback system is also required. But even if it were available, it is unclear how two complementary connection systems (direct and reverse) would exchange information about the weight value in them. This problem is called the *weight transport problem* (Grossberg, 1987). The second issue of backpropagation is *update locking problem* (Czarnecki et al., 2017) that requires to store activation values from the forward pass for the backward pass.

An alternative to backpropagation is learning methods based on the principle of locality. In the brain the synaptic weight can be changed only on the basis of some activity characteristics of neurons linked by this synapse and maybe by some global signal (Pfister and Gerstner, 2006; Gerstner et al., 2018). In the case of SNNs, the laws of synaptic plasticity used for learning are quite diverse, but many of them are modifications of the so-called Spike Timing Dependent Plasticity (STDP) (Sjöström et al., 2010). In the STDP model, the synapses that received spikes shortly before the neuron generated the spike, are strengthened, while the synapses that received spikes after the neuron generated the spike are weakened. Also there were attempts to develop local learning algorithms for ANNs by solving the weight transport problem (Lillicrap et al., 2016; Nøkland, 2016; Ororbia and Mali, 2019), the update locking problem (Mostafa et al., 2018; Nøkland and Eidnes, 2019) or both of them (Frenkel et al., 2021b). Local learning reduce the amount of global data transfer operations during learning, making it possible to train networks of almost unlimited size.

### 2.7. Sparsity

As known (Shoham et al., 2006; Quian Quiroga and Kreiman, 2010), less than 10% of neurons in the brain are usually active simultaneously. This is very different from the inference mode of classical neural networks, in which all neurons participate in calculations. This is determined by three factors.

The first factor is a high similarity of the subsequent frames. The transmission of the changed part of the signal only allows to decrease traffic dramatically, making the data sparse in time (temporal sparsity). For example, in computer vision, instead of transmitting information about each pixel of an image every tick of time, it is possible to transmit only the events of changing the intensity of specific pixels. This approach is used in event-based cameras like Dynamic Vision Sensors (DVS) (Gallego et al., 2020) that can generate an output signal immediately in a spike form.

The second factor is the threshold value of the membrane potential. Below the threshold, the neuron is silent even in the presence of an input signal. The resulting sparsity in data streams is called spatial (spatial sparsity). A similar idea is implemented in the Rectified Linear Unit (ReLU) activation. A significant number of neurons have an output equal to zero but, when computing on the GPU, these zeros will be multiplied anyway like other numbers.

The third factor is the sparseness of the graph of neural connections. There are no fully connected layers in the biological brain. Each neuron has a rather limited number of connections (~5,000). The sparsity of the data flow, conditioned by the network topology, is called structural (structural sparsity). For example, as shown in Frankle and Carbin (2018), in deep networks it is possible to zero out more than 90% of the weights of connections without degrading network performance.

### 2.8. Analog computing

Digital representation and information processing reveal the potential of numerical methods. However, in terms of the number of computational elements, this approach is expensive. An alternative approach is analog circuits. In AI systems, analog circuits can be used for two purposes: modeling the dynamics of the membrane potential and modeling synaptic operations. Let us consider them in more detail.

The behavior of biological neurons is usually modeled by a system of differential equations describing the membrane potential dynamics and the operation of ion pumps. In the absence of an analytical solution, the numerical solution of such a system of equations can be very costly. In the brain, neurons do not contain nodes that implement digital computations. They realize their function with the help of analog computations (membrane potential dynamics). But there are other physical objects that demonstrate similar dynamics (for example, an RC circuit). Thus, a biological neuron can be modeled not only by numerically solved differential equations, but also by using a suitable analog circuit described by such equations. Analog neurons can be 10,000 times faster and more energy efficient (Schmitt et al., 2017), and also they naturally support parallelism. The fundamental disadvantage of analog neurons is the difficulty in configuring and debugging them. Comparing analog implementations with digital ones, we note that analog neurons implement the “one neuron—one computer” principle, while in digital devices one computing unit usually models many neurons by switching the context between them (time-multiplexed neurons).

Another area where analog circuits are used is the implementation of synaptic operations. For example, the classical model of a neuron requires the computation of Multiply And Accumulate (MAC) operations that take the form: *sum* = *W*_1_*X_1_+...+*W*_n_*X_*n*_). It can be represented as a combination of Ohm's and Kirchhoff's laws: *sum* = *I*_1_*R_1_+...+*I*_n_*R_*n*_, where current I plays the role of signal X, and resistance R expresses the value of weight W. In such a circuit, all elements of the multiply-accumulate operation are performed absolutely in parallel in one clock cycle.

### 2.9. In-memory computing

When performing neural network emulation on the CPU/GPU, one core models a large number of neurons, sequentially switching context between them. This creates a significant time and energy overhead for transferring neuron context values to memory and back. There are no similar mechanisms observed in biological neurons.

A biological neuron implements the principle of in-memory computing. A biological neuron is simultaneously a device that stores its state (memory represented by the membrane potential and the strength of synaptic connections), and a device that performs computations (Sterling and Laughlin, 2015). This approach is free of von Neumann's limitations rooted in physical separation of the shared memory and the processors. The principle of in-memory computing dictates that the memory of a neuron is isolated from the other neurons. This principle implies the “one neuron—one computer” model, which is inherent to analog implementations of a neuron. However, in digital implementations, this approach is too wasteful since there is the possibility of modeling many neurons by one core due to context switching. That is why a hybrid approach is useful in digital implementations. The memory, located physically close to the computing core, is shared by a group of neurons that are modeled by that core (near-memory computing).

The Static Random Access Memory (SRAM) memory used for such solutions is more expensive in comparison to DRAM, and this limits the development of SRAM-based chips.

## 3. Neuromorphic projects

In the field of neuromorphic computing there is still no consensus on what properties should be copied from the brain. Next, we consider several existing projects and approaches that can be called neuromorphic based on the classification defined in the previous section. During the more than 35 years of the neuromorphic field there were many projects both in industry and in academia. Here we take into account only the most popular of them, to illustrate how our classification principle works.

### 3.1. TrueNorth

The TrueNorth project (Merolla et al., 2014) (2014, IBM), created under the auspices of the DARPA SyNAPSE program, is the world's first industrial neuromorphic chip.

The TrueNorth chip is digital, but it does not include general-purpose computational cores. The chip contains 4,096 neural cores, each of which simulates 256 firing neurons in real time and contains about 100 Kbits of SRAM memory for storing the state of synapses. A digital data bus is used for communication between neurons. Spikes are represented as Address Event Representation (AER) packets containing the identifier of the emitting neuron and the generation time. Multiplication and division are not supported in the digital circuits of TrueNorth neural cores. Only addition and subtraction are supported. The functioning of the neural core is not programmable. It is realized in the form of digital operations fixed at the hardware level.

Each neural core has 256 common inputs that can be arbitrarily connected to 256 neurons modeled in one core, i.e., one neuron cannot have more than 256 synapses. Moreover, the weight of each synapse is coded by 2 bits. This means that if neurons have excitatory and inhibitory synapses, the weight of synapses of each kind within one neuron can be only equal to one value. Such primitive coding scheme does not allow any learning algorithm to be realized directly on the chip.

TrueNorth is suitable for the execution of Convolutional (CNN) and Recurrent Neural Networks (RNN) (Merolla et al., 2014), but only in the inference mode. Another hardware platform (most frequently, GPU) should be used for the learning, after which the learned weights should be translated into a TrueNorth neurons configuration.

As an example of TrueNorth use, the world's first event-based gesture recognition system was demonstrated at CVPR 2017 (Amir et al., 2017). It consisted of a DVS camera and a TrueNorth chip, capable of recognizing 10 gestures with 96.5% accuracy in 0.1 s of gesture demonstration with a consumption of 0.18 W.

A year later, at CVPR 2018 (Andreopoulos et al., 2018), the same team presented an event-based stereo vision system, already consisting of two DVS cameras and eight TrueNorth chips, capable of determining the depth of a scene at 2,000 disparity maps per second, while remaining 200 times more energy efficient than other state-of-the-art solutions.

In 2019 (DeBole et al., 2019), they demonstrated a scene-understanding application that detects and classifies multiple objects in high definition aerial video at a throughput exceeding 100 frames per second.

### 3.2. Loihi

The Loihi project (2018, Intel) (Davies et al., 2018) was the first neuromorphic chip with on-chip learning. A Loihi chip includes 128 neural cores, three Pentium processors, and four communication modules for AER packet exchange. Each neural core simulates up to 1,024 spiking neurons and contains 128 Kbyte of SRAM to store the state of the synapses. Thus, the chip simulates approximately 128,000 neurons and up to 128,000,000 synapses. Transmission from neuron to neuron of all spikes is guaranteed, and if the flow of spikes becomes too intensive, the system simply slows down.

Synaptic weights can be from 1 to 9 bits and are dynamically modified, making it possible to learn directly on the chip. Besides the weight, the state of each synapse is described by a synaptic delay of up to 6 bits and some variables occupying up to 8 bits, which can be used as an auxiliary variable in the plasticity law. Local learning is realized by the procedure for recalculating the weights using the formula specified when configuring the core. The formula consists only of addition and multiplication operations.

A number of neurocomputers of different capacities have been created based on Loihi. Pohoiki Springs is the most powerful among them. The system includes 768 Loihi chips combined into 24 modules that are positioned on one motherboard, thus simulating 100,000,000 neurons.

More than a hundred scientific groups around the world use Loihi in research and applied problems (Davies et al., 2021), for example, for recognition and segmentation of images and smells, processing data sequences, realization of a proportional integral differential controller (PID) based on a spiking network, finding the shortest paths in a graph, and others. Some problems are solved by converting the trained classical neural networks into the SNN form. In other projects, SNNs are trained by using a surrogate gradient. At last, in several problems, local learning rules are applied. For example, local learning rules are used to control the robot arm (DeWolf et al., 2020) and copter balancing (Stagsted et al., 2020).

Intel announced the creation of the second version of the Loihi chip (xxx, 2021) in 2021. One Loihi 2 chip still contains 128 neural cores, simulating 120,000,000 synapses and 1,000,000 programmable (rather than configurable) neurons. The chip is built using Intel4 7nm technology, contains 2.3 billion transistors and has an area of 31 mm^2^. Another feature of Loihi 2 is 3D multi-chip scaling, i.e., the possibility of combining multiple chips into one system in a 3D (rather than 2D) space, thereby providing lower overheads for communication between the chips.

Loihi 2 realizes a generalized event-based communication model based on local broadcasts and graded spikes (that is, non-binary spikes), in which the spike value is coded by up to 32 bits. In this model, the spikes generated in the system can have amplitude, making it similar to NeuronFlow (considered below).

Alongside Loihi 2, Intel researchers introduced the Lava framework (xxx, 2022). It is a cross-platform, open-source framework that offers a new paradigm for describing process-based computing. Lava-has implementations for CPU, GPU, and Loihi 2.

### 3.3. Tianjic

The Tianjic project (2019, Tsinghua University) (Pei et al., 2019) is the first hybrid chip that can work effectively with both ANNs and SNNs. This possibility comes from the reuse of the same parts of circuits to work with different types of neural networks. The additional overhead for such versatility is only 3% of the chip area. Thus, using the Tianjic chip, it is possible to combine architectures of neural networks of different nature (ANN and SNN) within one system. One Tianjic chip contains 156 neural cores, simulating 40,000 neurons and 10,000,000 synapses. Each core contains 22 Kbyte of SRAM. The digital data bus is used for communication between the cores, and the signals are represented as AER packets. Scaling is achieved by combining chips into a 2D mesh network. On-chip learning is not supported. The neural network must be pre-trained on another platform (most frequently, GPU) and translated into the Tianjic configuration to work in the inference mode. Running SNN on Tianjic is 22 times faster and 10,000 times more energy efficient than on GPU. For ANNs, the gains are also significant:

- LSTM networks are 467 times more energy efficient,
- MLPs are 723 times more energy efficient and 35 times faster in terms of frame rates,
- CNNs are 53 times more energy efficient and 101 times faster in terms of frame rates.

An example of using Tianjic chip to create a bicycle motion control system is presented in Pei et al. (2019). This system, implemented on only one Tianjic chip, includes real-time object detection (CNN), object tracking (CANN), voice control (SNN), obstacle avoidance, and balance control (MLP). Another SNN, called a Neural State Machine (NSM), was used to integrate neural networks with each other.

### 3.4. SpiNNaker

The SpiNNaker project (2011, The University of Manchester) (Furber et al., 2014) was the first hardware platform designed exclusively for SNN research. The second generation of the platform SpiNNaker 2 (2018, Dresden University of Technology and The University of Manchester) (Höppner et al., 2021) is being developed as part of the European Human Brain Project.

SpiNNaker is not a chip—it is a massively parallel computer. Its main component is a specially designed microcircuit that has 18 Mbyte of SRAM and 144 ARM M4 microprocessors. These microprocessors have a very limited set of instructions (for example, they do not support division), but they have high performance and low power consumption. The second generation SpiNNaker added support for rate-based DNN, accelerators for numerical operations (exp, log, random, mac, conv2d) and dynamic power management.

Chips are mounted on boards with 56 chips per board. The boards are mounted into racks of 25 in each rack. The racks are combined into cabinets of 10 in each cabinet. All this, together with the control computer, makes up the 106 processor SpiNNaker neurocomputer (Mayr et al., 2019).

The operation of nodes within the entire computing system is asynchronous in relation to each other. This gives the entire system more flexibility and scalability but leads to the necessity of using AER packets for spike representation. Different communication strategies may be used (multicast, core-to-core, nearest neighbor).

With SpiNNaker, researchers can solve the problem of modeling the biological brain structures. The real-time simulation of a 1 mm^2^ cortical column (77,000 neurons, 285,000,000 synapses, 0.1 ms time-step) was demonstrated in Van Albada et al. (2018), while the best result of this benchmark on the GPU is two times slower than real time. Thanks to the asynchrony of SpiNNaker, modeling of a 100 mm^2^ column, instead of a 1 mm^2^ one, can be achieved by simply increasing the number of computational modules in the system, which is already unattainable for the GPU due to synchronization limitations.

### 3.5. BrainScaleS

The BrainScaleS project (2020, Heidelberg University) (Grübl et al., 2020) is an ASIC device developed as part of the European Human Brain Project. The main idea of BrainScaleS is to emulate the work of spiking neurons by applying analog computations. Electronic circuits are used for analog computations. Such electronic circuits are described by the differential equations resembling the equations expressing the behavior of membrane potential in biological neurons. One electronic circuit with a resistor and a capacitor corresponds to one biological neuron.

The first version of BrainScaleS was released as early as 2011, but it did not allow on-chip learning. In the second version, several digital processors were added to support local learning (STDP), in addition to the block of analog neurons. The digital data bus is used for communication between neurons using spikes in the form of AER packets. One chip can emulate 512 neurons and 130,000 synapses. The studies (Schmitt et al., 2017; Wunderlich et al., 2019) showed that a BrainScaleS neuron could work 10,000 times faster than a biological neuron in the analog implementation. Besides SNN emulation, BrainScaleS can be useful with classical ANNs, performing a matrix-vector multiplication operation in the analog mode.

The main disadvantage of the analog model of a neuron, based on an electrical circuit, is its inflexibility, i.e., the impossibility of changing the neuron model. The relatively large size of the analog neuron is another significant drawback. Works (Stradmann et al., 2021; Cramer et al., 2022) give examples of applying BrainScaleS to solve the problems of handwritten digit recognition (MNIST), speech recognition by SNN, and also a number of problems in the domain of ANN. For instance, for the spiking MNIST dataset, the classification accuracy was 97.2% with a latency of 8 μs, a dissipation of 2.4 μJ per image, and total consumption of 0.2 W for the entire chip connections. The learning was on-chip, but the surrogate gradient methods were used (Cramer et al., 2022). The paper (Schreiber et al., 2020) demonstrates the possibility of local learning for BrainScaleS in the Reinforcement Learning tasks using the R-STDP algorithm. The system was trained to control a slider bar in a computer game similar to Atari PingPong.

BrainScaleS is not the only ASIC for simulating analog neurons: the NeuroGrid project (Benjamin et al., 2014) (2009, Stanford University) was based on the same idea. However, it was decided to exclude it from this review because the project seems to have been abandoned (the latest updates were in 2014).

### 3.6. NeuronFlow

The NeuronFlow project (Moreira et al., 2020) (2020, GrAI Matter Labs) presented the GrAIOne chip. The project implements the idea of creating an accelerator to speed up sparse computations and to deal with event-based data. The chip is capable of accelerating both ANN and SNN but it does not support on-chip learning.

GrAIOne contains 196 neural cores simulating 200,704 neurons. Each core contains 1,024 neurons and SRAM for storing the state. The cores communicate via the digital data bus using AER packets.

The term NeuronFlow denotes an architecture with the underlying idea to speed up computations by using a high correlation of frames in data-flow processing tasks (audio, video). For example, each next frame differs only a little from the previous one in a video. Therefore, most neuron activations for the two consequent frames will also be very similar. Then it is possible to avoid sending activation from one neuron to another if it has not changed significantly from what it was at the previous step. This approach gives an opportunity to drastically reduce the number of synaptic operations (multiplications of weights by input values) and memory access operations. Thus, the NeuronFlow architecture is suitable only for processing slowly changing data; otherwise, its advantages are canceled out.

The paper (Khoei et al., 2020) demonstrates the optimization of the PilotNet neural network operation by reducing the number of floating point operations by 16 times. PilotNet is Nvidia's architecture for controlling the steering wheel of an unmanned vehicle. The network receives an image from the front view camera as an input and calculates the steering wheel angle.

### 3.7. DYNAP

DYNAP (Dynamic Neurormorphic Asynchronous Processors) is a family of solutions from SynSence, a company from the University of Zurich. The company has a patented event-routing technology for communication between the cores.

According to Moradi et al. (2017), the scalability of neuromorphic systems is mainly limited by the technologies of communication between neurons. All other limitations are not so important. Researchers at SynSence invented and patented a two-level communication model based on choosing the right balance between point-to-point communication between neuron clusters and broadcast messages within clusters. The company has presented several neuromorphic processors (ASICs): DYNAP-SE2, DYNAP-SEL, and DYNAP-CNN.

The Dynap-SE2 and Dynap-SEL chips are not commercial projects and are being developed by neuroscientist as tools for their research. But Dynap-CNN (2021 tinyML) is marketed as a commercial chip for efficient execution of CNNs converted to SNNs. Whereas the Dynap-SE2 and Dynap-SEL research chips implement analog computing and digital communication, Dynap-CNN is fully digital.

Dynap-SE2 is designed for feed-forward, recurrent and reservoir networks. It includes four cores with 1k LIFAT analog spiking neurons and 65k synapses with configurable delay, configurable weight and short term plasticity. There are four types of synapses (NMDA, AMPA, GABAa, GABAb). The chip is used by researchers for exploring topologies and communication models of the SNN.

The main distinctive features of the Dynap-SEL chip are the support for on-chip learning and large fan-in/out network connectivity. It has been created for biologically realistic networks emulation. The Dynap-SEL chip includes five cores. But only one core has plastic synapses. The chip realizes 1,000 analog spiking neurons and up to 80,000 configurable synaptic connections, including 8,000 synapses with integrated spike-based learning rules (STDP). Researchers use the chip to model cortical networks.

The Dynap-CNN chip is available with the Development Kit since 2021. Dynap-CNN is a 12 mm^2^ chip, fabricated in 22 nm technology, hosting over one million spiking neurons and four million programmable parameters. Dynap-CNN is completely digital and realizes a linear neuron model without leakage. The chip is best combined with event-based sensors (DVS) and is suitable for image classification tasks. In the inference mode the chip can run a SNN converted from a CNN, in which there may be no more than nine convolutional or fully connected layers and not more than 16 output classes. On-chip learning is not supported. The original CNN must be initially created with PyTorch and trained by classical methods (for example, on GPU). Further, using the Sinabs.ai framework (an open source PyTorch based library), the convolutional network can be converted to a spiking form for execution on Dynap-CNN in the inference mode.

Dynap-CNN has demonstrated the following results:

- CIFAR-10: 1mJ at 90% accuracy,
- Attention detection: less than 50 ms and 10 mW,
- Gesture recognition: less than 50 ms and 10 mW at 89% accuracy,
- Wake phrase detection: less than 200 ms at 98% sensitivity and false-alarm rate less than 1 per 100 h (office background).

### 3.8. Akida

Akida (Vanarse et al., 2019) is the first commercial neuromorphic processor, commercially available since August 2021. It has been developed by Australian BrainChip since 2013. Fifteen companies, including NASA, joined the early access program. In addition to Akida System on Chip (SoC), BrainChip also offers licensing of their technologies, providing chip manufacturers a license to build custom solutions.

The chip is marketed as a power efficient event-based processor for edge computing, not requiring an external CPU. Power consumption for various tasks may range from 100 μW to 300 mW. For example, Akida is capable of processing at 1,000 frames/Watt (compare to TrueNorth with 6,000 frames/Watt). The first generation chip supports operations with convolutional and fully connected networks, with the prospect to add support of LSTM, transformers, capsule networks, recurrent and cortical neural networks. ANN network can be transformed into SNN and executed on the chip.

One Akida chip in a mesh network incorporates 80 Neural Processing Units, which enables modeling 1,200,000 neurons and 10,000,000,000 synapses. The chip is built at TSMC 28 nm. In 2022, BrainChip announced the second generation chip at 16 nm.

Akida's ecosystem provides a free chip emulator, TensorFlow compatible framework MetaTF for the transformation of convolutional and fully connected neural networks into SNN, and a set of pre-trained models. When designing a neural network architecture for execution at Akida, one should take into account a number of additional limitations concerning the layer parameters (e.g., maximum convolution size is 7, while stride 2 is supported for convolution size 3 only) and their sequence.

The major distinctive feature is that incremental, one-shot and continuous learning are supported straight at the chip. At the AI Hardware Summit 2021 BrainChip showed the solution capable of identifying a human in other contexts after having seen him or her only once. Another product by BrainChip is a smart speaker, that on having heard a new voice asks the speaker to identify and after that calls the person by their name. There results are achieved with help of a proprietary local training algorithm on the basis of homeostatic STDP. Only the last fully connected layer supports synaptic plasticity and is involved in learning.

Another instructive case from the AI Hardware Summit 2021 was a classification of fast-moving objects (for example, a race car). Usually, such objects are off the frame center and significantly blurred but they can be detected using an event-based approach.

### 3.9. Mythic

Mythic project was started in 2012 at the University of Michigan. In 2020 Mythic Inc. presented the M1076 Analog Matrix Processor (AMP) based on flash memory. The main idea is to use analog computing where computation is performed directly inside the memory array itself. This is possible by using the memory elements as tunable resistors, supplying the inputs as voltages, and collecting the outputs as currents. This allows it to achieve 3-4 watt when running typical complex models in a single chip.

Although Mythic AMP does not support SNNs and learning at all, we include this DataFlow based chip in the review because it uses two fundamental neuromorphic approaches—Compute-in-Memory, and Analog Computing.

The Mythic AMP delivers up to 25 Tera Operations per Second (TOPS). The first generation of Mythic AMP uses 40 nm complementary metal-oxide-semiconductor (CMOS) technology. Single chip integrates 76 AMP tiles to store up to 80M weight parameters and execute matrix multiplication operations without any external memory (Mythic, 2020).

Each tile has a large Analog Compute Engine (ACE) to store bulky neural network weights, local SRAM memory for data being passed between the neural network nodes, a single-instruction multiple-data (SIMD) unit for processing operations not handled by the ACE, and a 32-bit RISC-V nano-processor for controlling the sequencing and operation of the tile. The tiles are interconnected with an efficient on-chip router network, which facilitates the dataflow from one tile to the next.

The Mythic AI workflow includes optimization and compilation. Neural network models developed in standard frameworks such as Pytorch and TensorFlow are optimized, quantized from 32 bit floating point to 8 bit integer, and then retrained. Resultant model are then programmed into the Mythic AMP for inference. In addition, Mythic provides a library of pre-qualified models for the most popular AI use cases, including YOLOv3, YOLOv5, ResNet-50, ResNet-18, SegNet, and OpenPose Body25.

## 4. Memristors

The majority of the neuromorphic hardware systems discussed above are based on existing CMOS technology. There is no direct similarity at the level of physical mechanisms between CMOS devices and elements of biological neural networks. Because of this, CMOS devices can only numerically simulate biological neural networks.

The interest in neuromorphic systems that follow the rules of biological learning has prompted to explore alternative technologies closer to the biological prototypes. Currently, the most mature technology of this kind is memristors. A memristor is a two-terminal device capable of changing its conductivity depending on the voltage/current applied to the terminals. Such an element was theoretically predicted in 1971 (Chua, 1971), and its practical existence was experimentally demonstrated in 2008 (Strukov et al., 2008). In subsequent years, it was discovered that many materials, mainly at the nanometer scale, can exhibit memristive properties with different physical mechanisms of conductivity switching (Camuñas-Mesa et al., 2019). A large variety of materials exhibiting memristic properties are also classified according to the physical effect: magnetoresistive memory effects, ferroelectric effects, electrostatic effects, electrochemical metallization cell, valence change effect, thermochemical effect, phase change memory, nanomechanical memory (Kang, 2021).

Now, there are two main directions of memristor usage in neuromorphic applications: vector-matrix multiplication in memory and spiking neural networks.

### 4.1. Vector-matrix multiplication in memory

Main operations of classical neural networks, built upon CMOS technology, are as follows: multiplication, addition and activation function computation. Weights of neural networks are generally stored in SRAM or DRAM cells. CMOS circuits are scalable but the available scalability is still not enough for many neural network applications. Besides, SRAM cell size is too large for high-density integration, while DRAM cells require periodical refreshing to prevent data loss. In neural computation, it is frequently needed to extract data from the memory, transfer data to the computing core, perform computations, and then send the results to the memory through the same data bus. Such an operation sequence being applied to a large amount of data stored in the memory causes a significant computation speed limitation and large power consumption. This factor substantively limits the efficiency of the deep learning technique in the field of big data (Xia and Yang, 2019).

**Figure 4:**
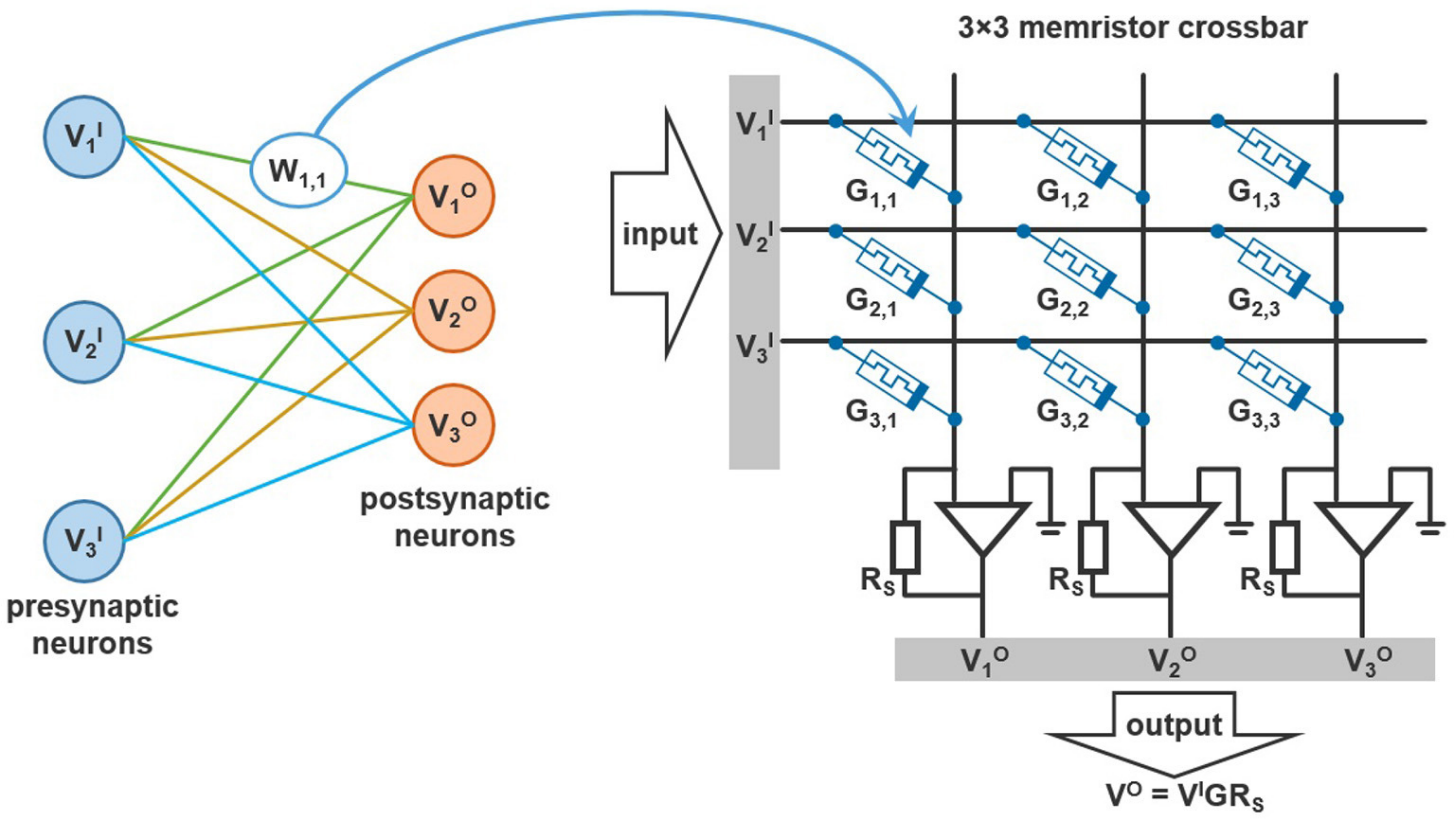
Typical 3 × 3 memristor crossbar used in neuromorphic applications.

Memristor crossbar circuits make it possible to combine addition, multiplication and data storage in a single element. A crossbar is a junction of conducting wires, placed perpendicularly to each other, with memristors positioned at the intersections (see Figure 4). As it is seen, data are processed and stored in the crossbar. It leads to saving chip space and achieving very low energy consumption.

Researchers have developed various topologies and training algorithms for memristor based neural networks. In the study (Hu et al., 2014), it was demonstrated a one-layer neural network with a 128 × 64 memristor massive. During the experiment, an image recognition accuracy of 89.9% accuracy was achieved for the MNIST dataset. The study (Li et al., 2019) demonstrated a two-layer RNN based on 14 memristor LSTM blocks. This network achieved 79.1% accuracy for the task of a human walking classification on the USF-NIST data set.

At the moment, neuromorphic memristor computing systems have not yet reached production ready state, but we would like to note several significant research projects demonstrating characteristic neuromorphic properties in memristor approaches.

- The memristor project chip of Tsinghua University's Laboratory for Emerging Memory and New Computation (LEMON). In their key work (Yao et al., 2020), the authors conduct an experimental demonstration of a neuromorphic memristor chip focused on MAC operations with high energy efficiency. The chip uses TiN/TaOx/HfOx/TiN memristor cells in a 1T1R circuit (one transistor, one memristor) as the element base. The chip is an array of 2048 1T1R elements connected in a crossbar architecture. Further, to obtain a multilayer neural network, multiple chips were placed on a common printed circuit board with additional field-programmable gate array components, to control multiple memristor chips.
  The work successfully demonstrated a complete five-layer memristive CNN for digital image recognition with the MNIST dataset. As a consequence of a small set of levels of memristor conductivity, a transition of data representation from a 32-bit floating-point type to a 15-level fixed-point type was performed. With the proposed hybrid learning scheme, the experimental recognition accuracy reached 96.19%. In addition, the replication of convolutional kernels to three parallel memristor convolvers was implemented to reduce the latency of the memristor CNN by about a factor of 3. A performance test of the memristor-based neuromorphic computing system shows 110 times higher energy efficiency (11014 GOPS/Watt) and 30 times better performance density (1164 GOPS/mm^2^), compared to the Tesla V100 GPU.
- A project by researchers at the University of Massachusetts. In their pivotal paper (Li et al., 2018a), the authors experimentally demonstrate *in situ* learning in a multilayer neural network on a memristor component base. As the basis of the component base, they used recently developed Ta/HfO2/Pt memristors to achieve stable tunable multilevel behavior with a linear current-voltage (IV) relationship (Jiang et al., 2016). Each memristor was connected to a 1T1R series transistor (one transistor, one memristor). The chip contained a 128 × 64 array of 1T1R elements, connected by crossbar architecture. The speed and reliability of 1T1R circuit and the two-pulse circuit conductivity update, proposed by the authors, make it possible to train the network *in situ*.
  Here, the network was trained using stochastic gradient descent to classify handwritten digits in the MNIST dataset. For each new sample of training data, the network first performs inference to get the log-probability of the label for each output by the softmax function, and then the weights in each layer are updated accordingly. The backward error propagation in this work is calculated programmatically from the values of the read weights. In the future, back propagation can be implemented inside a memristor rod by applying a voltage vector representing the current layer error to the lower electrodes of the rod and reading the current vector from the upper electrodes for the previous layer error. After using the entire training database (80,000 handwritten digit images), the network correctly classified 91.71% of the 10,000 images in the separate test set.

### 4.2. Spiking neural networks

Hardware demonstrations of SNNs with the use of memristor devices have mostly focused on the unsupervised learning. Synaptic weights change in accordance with the biologically realistic STDP rule. It was experimentally shown that if appropriate signal forms are used then memristor devices can show the weight adaptation behavior similar to STDP (Li et al., 2018b).

In addition to demonstrations of neuromorphic properties on single memristors, one can highlight a project demonstrating computational capabilities on an array of spiking memristor elements:

- IBM's memristor-based neuromorphic core project. In their key work (Kim et al., 2015), the authors demonstrate a neuromorphic core with a phase change memory (PCM) synaptic matrix that changes its physical properties when heated, with the nature of the change in physical properties depending on the heating dynamics. The neuromorphic core consists of 64 thousand cells (256 axons per 256 dendrites) with *in situ* learning capability. 256 configurable on-chip neural circuits perform leaky integration and fire neurons and synaptic weight update based on STDP. The 2T1R (two transistors, one PCM memristor) unit cell design separates the LIF pathways and the STDP learning pathways, minimizing neural circuit size. The circuit implementation of the STDP learning algorithm, together with the 2T1R structure, allows learning asynchronously and simultaneously within an array, avoiding the additional complexity and power consumption.

Other studies based on a selection of switching mechanisms and dynamic parameters of memristors demonstrated different basic neuromorphic principles such as: symmetric and asymmetric plasticity, spike-rate-dependent plasticity, long-term depression and long-term potentiation. Their implementations are described in Basu et al. (2018). The memristor technologies were also used in hardware implementations for the Hodgkin–Huxley, Morris–Lecar, and FitzHugh–Nagumo neuron models. Their implementations are given in Sung et al. (2018).

Speaking of the general properties of memristor materials and structures built on their basis, the following main characteristics valuable for the neuromorphic approach should be noted:

- When a current flows through a memristor, there is a change in its physical structure, which leads to a change in its conductivity. This change of the element itself differs from existing charge-storage-based memory cells (DRAM, SRAM, Flash, etc.) by its significantly longer state retention duration. Based on this property, the development of non-volatile resistive random access memory (ReRAM) is underway, which will have: an extended data storage lifetime (>10 years), low operating voltage (<1 V), a large number of rewrite cycles (>10^17^ cycles), low power consumption (10 fJ/bit) (Mehonic et al., 2020).
- Memristors can be used both in fully digital (binary) and analog modes. The manifestation of analog properties is the ability to set a fixed conductivity in a continuous range of values. Using the analog property it is possible to get an element that is able to store information in a multilevel mode of conduction states. The number of states in modern memristive structures reaches 256, which corresponds to 8 bits.
- The conductivity of the memristor depends on the total value and direction of the current passing through it. This ability allows us to consider the memristor as an element that has a memory of the value of the passed current.
- Memristor operation timescale may vary from second to nanoseconds.
- Memristors can be scaled down to less than 10 nm and made compatible with existing CMOS technology to achieve high computational density (Zahoor et al., 2020).

Memristors have unique properties, the main of which is the presence of neuromorphic properties at the elementary level. Their unique properties have attracted the interest of both small research laboratories and large international companies such as IBM, HP, Intel, Samsung, etc. (Shukla and Sharma, 2017). At the moment there are several projects around the world that develop the topic of memristor systems and allow for a relatively low cost to buy a chip with a set of memristors. The most popular such project is Knowm (Knowm, 2015).

Although worldwide interest in memristor devices is high, there are currently a number of unresolved issues in memristor technology that are limiting factors for the mass distribution of off-the-shelf high-performance neuromorphic devices. These limiting factors include: variability in the parameters produced by memristors; non-linearity of current-voltage characteristics; limited conductivity range (Im et al., 2020) and the problem of sneak current paths, which currently leads to the need for additional logic elements next to each memristor (Zidan et al., 2013).

## 5. Conclusion

In conclusion, we classify neuron modeling methods, compare the considered chips, and highlight the existing trends and limitations that hinder the development of neuromorphic technologies.

It is convenient to separate the modeling of the body of a neuron (Table 2) and its synapses (Table 1).

**Table 1:** A comparison of neuromorphic approaches to synapses modeling.

**Approach**	**Network**	**In-memory computation**	**Signal**	**On-device training**
Computational modeling on digital logic	ANN SNN	No/near-memory	Digital	Backprop Surrogate Gradient/STDP
Analog modeling (Memristors)	ANN	Yes	Analog	- STDP

Table 2 compares synapse modeling approaches. In general, synapses can be modeled both with digital circuits and in an analog way. Both of these modeling approaches are suitable for both ANNs and SNNs. However, among all the considered projects on digital logic, none of them allows performing calculations in memory. They require data exchange between Arithmetic Logic Unit (ALU) and memory cells. One way to mitigate the von Neumann problem of data exchange is to move the memory closer to computing by using more SRAM memory. Many projects like Loihi, TrueNorth, Tianjic, Neuronflow use this approach. In turn, analog synaptic computing is an example of in-memory computing.

**Table 2:** A comparison of neuromorphic approaches to neuron soma modeling.

**Approach**	**Network**	**In-memory computation**	**Signal**
Computational modeling on digital logic	ANN SNN	No/near-memory	Digital
Analog modeling (RC circuit)	SNN	Yes	Analog

On digital logic, we can implement almost any learning algorithm, moreover, we can leave ample opportunities for its customization. Therefore, we see implementations of both the classic Backprop (in CPU/GPU/TPU), and various STDP variants (Loihi), as well as special variants of surrogate gradient for spiking networks (Loihi). In analog devices (like memristors) that are designed for SNN networks, we see the implementation of some STDP-like rules due to its physical nature.

Table 1 compares approaches to neuron body modeling. There are two main ways to model the body of a neuron: using digital logic and using analog RC circuits. The approach based on digital logic is suitable for modeling both ANNs and SNNs, while the RC circuit approach is only suitable for modeling SNNs. As well as a similar digital version for synapses, it does not allow calculations in memory, at best it allows calculations near-memory. The approach on RC circuits is an analog alternative. It uses special analog calculators, which have dynamics similar to a neuron membrane. These circuits, as well as biological neurons, are both a computer and a state store, which allows us to speak of them as in-memory calculators.

Table 3 provides the comparison of all the projects discussed in the article. Analyzing this table, we come to the following conclusions:

**Table 3:** Comparison of neuromorphic chips.

**Chip/neural** ** computer**	**In-memory computation**	**Signal**	**Size neurons/synapses**	**On-device learning**	**Analog**	**Event-based**	**nm**	**Features**
CPU/GPU/TPU	No	Real numbers, spikes	-	Backprop/STDP	No	No	5	High popularity, rich ecosystem, advanced engineering technologies
TrueNorth	Near-memory	Spikes	1M/256M	No	No	Yes	28	First industrial neuromorphic chip without training (IBM)
Loihi	Near-memory	Spikes	128K/128M	STDP	No	Yes	14	First neuromorphic chip with training (Intel)
Loihi2	Near-memory	Real numbers, spikes	120K/1M	STDP, surrogate backprop	No	Yes	7	Development of Loihi ideas, non-binary spikes, neurons can be programmed
Tianjic	Near-memory	Real numbers, spikes	40K/10M	No	No	Yes	28	Hybrid chip with effective support of both SNN and ANN, energy efficiency
SpiNNaker	Near-memory	Real numbers, spikes	-	STDP	No	No	22	Scalable computer for SNN simulation
Brain-ScaleS	Yes	Real numbers, spikes	512/130K	STDP, Surrogate gradient	Yes, membrane	Yes	65	Analog neurons at RC circuits, large size
GrAIOne (Neuron- Flow)	Near-memory	Real numbers, Spikes	200K/	No	No	Yes	28	NeuronFlow architecture, effective support of sparse computations, support of ANN and SNN
DYNAP SE2, SEL, CNN	Near-memory	Spikes	1K/65K 1K/80K 1M/4M	STDP (SEL)	SE2, SEL	Yes	22	Proprietary communication protocol
Akida	Near-memory	Spikes	1,2M/10B	STDP (last layer)	No	Yes	28	First commercial neuromorphic processor with incremental, one-shot, and continuous learning for CNN
Mythic	In-memory	Real numbers	/80M	-	Yes	Yes	40	-
Memristor (Tsinghua University)	Yes	Real numbers	192/ 2048	No	Yes (15 signal levels)	Yes	500	CNN-optimized memristor chip, one chip contains 2048 1T1R elements
Memristor (Univ. of Massachusetts)	Yes	Spikes	192/ 2048	No	Yes	Yes	2 μm	128 × 64 memristor array according to 1T1R circuit
Memristor (IBM)	Yes	Spike	512/ 64k	Yes	Yes	Yes	50	2T1R design allows each synaptic cell to operate asynchronously in either LIF or STDP mode

Almost all projects are implemented on digital logic. Analog circuits are less flexible than digital circuits and suffer from the problem of computational instability and debugging problems.

There is a noticeable trend toward hybrid architectures that allow the transmission of non-binary (graded) spikes, which carry not only the fact of its presence, but also the numerical value. Tianjic and Neuroflow had this feature in the first versions, Loihi added it in the second generation. Despite the fact that this is a departure from the classical way of brain modeling, it makes it much easier to adapt modern ANN networks to these computer architectures.

Another notable trend is hybridization in terms of the possibility of executing both classical (ANN) and spiked (SNN) neural networks on a single chip. For example, Tianjic and NeuronFlow allow us to work not only with SNNs, but also with ANNs.

Next conclusion is that many neuromorphic processors rely on the sparseness of neuron activations in time. It allows saving energy and the number of message exchanges between neurons, which reduces the bandwidth requirements for the bus.

Next, despite the large number of new and diverse architectures that have emerged recently there are a large number of issues/areas that still require more detailed study. Despite the extremely active development of neuroscience in recent years, there is still no generally accepted theory of the brain functioning and consciousness. Many issues related to learning also remain open. There are many open questions about suitable neuron models, learning algorithms and topology of the network. Since we do not know the exact answers to these questions, we have to add more flexibility to the hardware, which increases the number of transistors per neuron. For example, SpiNNaker, due to the use of general-purpose cores, has more flexibility than Loihi or TrueNorth, but it consumes a much bigger number of transistors per neuron.

Next conclusion is that analog computers (e.g., BrainScaleS) can speed up bio simulations up to x10000 compared to SpiNNaker, Loihi, and TrueNorth, which perform simulations at a speed comparable to the speed of the brain. However, analog BrainScaleS is much less flexible than digital processors.

All of the above problems present developers with the challenge of finding a balance between limitations and desirable properties. To date, there is no single generally accepted style of architecture/list of styles of architecture. Research in this area continues.

Due to flexibility and the well-established manufacturing process of digital chips, neuromorphic processors apparently will remain digital in the coming years. They will be based on architectures similar to Loihi, where many computing cores are connected by a digital data bus. AER packets are transmitted over this bus to exchange information between the cores (Frenkel et al., 2021a), and local messages (within the cores) are propagated using a broadcast. The idea of storing the state and weights of neurons in SRAM memory seems to be temporary due to the fact that SRAM memory has a low density and is volatile. The latter means that we cannot turn off entire chip cores with resting neurons and turn them on only when they are needed. We look forward to new types of memory that solve this problem.

## Author contributions

DI, DL, MK, AC, and AG contributed to the conception and design of the study. DI and DL wrote the main part of the introduction and the second part of the article about neuromorphic approaches. DI, DL, and MK wrote the part with the descriptions of neuromorphic projects. AC and AG wrote the last part of the article about memristors. All authors contributed to manuscript revision, read, and approved the submitted version.

## Funding

This work was supported by the Ministry of Science and Higher Education of the Russian Federation (Grant No. 075-15-2020-801).

## Conflict of interest

DI, MK, and DL were employed by the company Cifrum. The remaining authors declare that the research was conducted in the absence of any commercial or financial relationships that could be construed as a potential conflict of interest.

## Publisher's note

All claims expressed in this article are solely those of the authors and do not necessarily represent those of their affiliated organizations, or those of the publisher, the editors and the reviewers. Any product that may be evaluated in this article, or claim that may be made by its manufacturer, is not guaranteed or endorsed by the publisher.

## Glossary

- AI - artificial intelligence
- RL - reinforcement learning
- NN - neural network
- DNN - deep neural network
- ANN - artificial neural network
- SNN - spiking neural network
- CNN - convolutional neural network
- RNN - recurrent neural network
- CANN - continuous attractor neural networks
- LSTM - long short-term memory
- MLP - multilayer perceptron
- STDP - spike-timing-dependent plasticity
- R-STDP - reward-modulated spike-timing-dependent plasticity
- ReLU - rectified linear unit
- MAC - multiply and accumulate
- MNIST - Modified National Institute of Standards and Technology
- CIFAR - Canadian Institute For Advanced Research
- CVPR - Conference on Computer Vision and Pattern Recognition
- CPU - central processing unit
- GPU - graphical processing unit
- TPU - tensor processing unit
- ASIC - application-specific integrated circuit
- FPGA - field-programmable gate array
- SoC - System on Chip
- RISC-V - reduced instruction set computer five
- CMOS - complementary metal oxide-semiconductor
- PCB - printed circuit board
- 1T1R - one transistor - one memristor
- RC - resistance-conductance circuit
- ALU - arithmetic logic unit
- HBM - high bandwidth memory
- RAM - random access memory
- DRAM - dynamic random access memory
- SRAM - static random access memory
- ReRAM -resistive random access memory
- SIMD - single instruction multiple data
- AER - address event representation
- AMP - analog matrix processor
- ACE - analog compute engine
- 2D - two dimensions
- 3D - three dimensions
- DVS - dynamic vision sensors
- TOPS - tera operations per second
- GOPS - giga operations per second
- MACGOPS - giga MAC operations per second
- HBP - Human Brain Project
- IBM - International Business Machines
- AMD - Advanced Micro Devices
- DARPA - Defense Advanced Research Projects Agency
- SyNAPSE - Systems of Neuromorphic Adaptive Plastic Scalable Electronics
- DYNAP - Dynamic Neurormorphic Asynchronous Processors
- NASA - National Aeronautics and Space Administration
- TSMC - Taiwan Semiconductor Manufacturing Company
